# Can Radiosensitivity Associated with Defects in DNA Repair be Overcome by Mitochondrial-Targeted Antioxidant Radioprotectors

**DOI:** 10.3389/fonc.2014.00024

**Published:** 2014-02-17

**Authors:** Joel S. Greenberger, Hebist Berhane, Ashwin Shinde, Byung Han Rhieu, Mark Bernard, Peter Wipf, Erin M. Skoda, Michael W. Epperly

**Affiliations:** ^1^Department of Radiation Oncology, University of Pittsburgh Cancer Institute, Pittsburgh, PA, USA; ^2^Department of Chemistry and Center for Chemical Methodologies and Library Development, University of Pittsburgh, Pittsburgh, PA, USA

**Keywords:** Fanconi anemia, radioprotectors, GS-nitroxide, clinical radiosensitivity, mitochondria

## Abstract

Radiation oncologists have observed variation in normal tissue responses between patients in many instances with no apparent explanation. The association of clinical tissue radiosensitivity with specific genetic repair defects (Wegner’s syndrome, Ataxia telangiectasia, Bloom’s syndrome, and Fanconi anemia) has been well established, but there are unexplained differences between patients in the general population with respect to the intensity and rapidity of appearance of normal tissue toxicity including radiation dermatitis, oral cavity mucositis, esophagitis, as well as differences in response of normal tissues to standard analgesic or other palliative measures. Strategies for the use of clinical radioprotectors have included modalities designed to either prevent and/or palliate the consequences of radiosensitivity. Most prominently, modification of total dose, fraction size, or total time of treatment delivery has been necessary in many patients, but such modifications may reduce the likelihood of local control and/or radiocurability. As a model system in which to study potential radioprotection by mitochondrial-targeted antioxidant small molecules, we have studied cell lines and tissues from Fanconi anemia (Fancd2^−/−^) mice of two background strains (C57BL/6NHsd and FVB/N). Both were shown to be radiosensitive with respect to clonogenic survival curves of bone marrow stromal cells in culture and severity of oral cavity mucositis during single fraction or fractionated radiotherapy. Oral administration of the antioxidant GS-nitroxide, JP4-039, provided significant radioprotection, and also ameliorated distant bone marrow suppression (abscopal effect of irradiation) in *Fancd2*^−/−^ mice. These data suggest that radiation protection by targeting the mitochondria may be of therapeutic benefit even in the setting of defects in the DNA repair process for irradiation-induced DNA double strand breaks.

## Introduction

The relative susceptibility of individual patients to acute and chronic side effects of therapeutic ionizing irradiation has been the subject of intense controversy (1). Radiation oncologists expect a greater degree of acute side effects as target volumes, radiation fraction size, and total dose increase, and overall treatment time is decreased (1). Concern for the parameters of acute tissue toxicity has led to innovative and valuable modifications of radiotherapy technology including intensity modulated radiotherapy, stereotactic radiosurgery, image-guided radiotherapy, and respiratory gating. These modalities have facilitated a decrease in overall tissue dose (integral dose) and are designed to reduce acute radiotherapy side effects. Most prominently, the use of brachytherapy techniques in which ionizing irradiation sources are implanted permanently or transiently into tissues greatly reduces total target dose. These principals have been applied to clinical radiation therapy for over 100 years (1).

Despite uniform approaches toward reducing normal tissue toxicity, there remain continual reports of variation between patients with respect to the likelihood of developing rapid normal tissue damage effects, symptomatology, and potential requirement for radiotherapy treatment breaks, reduced total dose, or reduced fraction size. The mechanism of the radiosensitivity of tissues in specific patients is not always available. Specific DNA repair defects have been associated with radiosensitivity. These include the genetic defects in ataxia telangiectasia, Bloom’s syndrome, Wegner’s syndrome, and Fanconi anemia (1–4). However, even within these rare disease categories, there remains heterogeneity with respect to expression of the phenotypic response of clinical radiosensitivity. In particular, Fanconi anemia is diagnosed by specific clinical attributes of short stature, abnormality of thumb morphology, café au lait spots, and other observable phenotypic changes, but these are not always present in FA patients (5–7). FA is then confirmed by sensitivity of cells to DNA cross-linking agents such as mitomycin C (6, 7). Given the complexity of the FA pathway, involving 15 or more proteins, the repair mechanism in this clinical syndrome has been termed one of a “defect in the scaffolding” of DNA repair process (6, 7). FA proteins serve as a base for the complex interaction of proteins in DNA double strand break repair. While two patients may be diagnosed with FA and in fact have defects at the level of the same protein (for example, FANC-A), one may be radiosensitive and the other may not (5). This observation is particularly important, because of the presence in FA patients of a high likelihood development of epithelial cancers, including head and neck cancer. There are many reports of FA patients who suffer significant morbidity of clinical radiotherapy, including inability to tolerate standard clinical fractionation for a 5.5 weeks course of post-operative radiotherapy for head and neck cancer (5). Given the same genotypic complement in FA patients with defects in the same protein of the same pathway, radiation oncologists have been cautious in treating FA patients, knowing that some will experience significant normal tissue toxicity. Furthermore, the use of radiotherapy in treatment of FA patients has been discouraged by many investigators in the field because of the likelihood of significant side effects (5).

## Materials and Methods

### Mice and animal care

Fanconi anemia (*Fancd2*^−/−^) mice were bred from the mating of *Fancd2*^+/−^ heterozygote pairs and were derived from either the C57BL/6 background (2) or the FVB/N background (3) strains. Mice were housed four per cage according to Institutional IACUC guidelines and fed standard Purina^®^ chow and deionized water. Animals for irradiation studies were uniformly 6–8 weeks of age and both males and females were studied.

### Animal irradiation

Total body irradiation was carried out using a cesium gamma cell irradiator at 70 cGy (8). Head and neck irradiation was carried out using a Varian Clinac 6 mV linear accelerator radiation beam and using a technique whereby thoracic cavity and abdomen as well as all four limbs were shielded such that only the head and neck were irradiated. All irradiation doses were calibrated using thermoluminescent dosimeters and dose rate was uniformly 200 cGy/min (9, 10).

### *In vitro* radiation studies

Bone marrow stromal cell lines from several mouse strains were derived from the adherent layer of long-term bone marrow cultures (11). Briefly, long-term bone marrow cultures were established from *Fancd2*^−/−^, *Fancd2*^+/−^, and *Fancd2*^+/+^ mice (C57BL/6J mouse strain). Non-adherent cells were removed weekly and assayed for total cells produced, as well as those cells capable of producing colonies in semi-solid medium in secondary culture. Colonies were scored at day 7 and day 14. The results of these studies showed significant decrease in the capacity of cultures to produce hematopoietic cells over 22 weeks (Permanent cell lines were established from the adherent layer of these long-term bone marrow cultures and produced cell lines that are adherent in culture and fibroblastic. These are also called mesenchymal stem cells.). Non-adherent cells from the 4-week harvest of long-term marrow cultures were carried in medium supplemented with IL-3 and clonal cell lines established. These are termed hematopoietic cells and are IL-3-dependent multilineage colony forming cells capable of forming neutrophils, macrophages, erythroid cells, megakaryocytes, and monocytes. Briefly, the adherent cell layer from 4-week-old continuous marrow cultures was trypsinized and cells were passaged weekly in Dulbecco’s modified Eagle’s medium supplemented with 10% fetal bovine serum and antibiotics according to published methods. After 10 weeks of passage, cell lines were cloned by limiting dilution technique in 96 well plates and using Poisson statistics. Single cell-derived clonal lines were passaged weekly according to published methods.

Interleukin-3 (IL-3)-dependent hematopoietic progenitor cell lines were derived from the non-adherent cells of 4-week-old long-term bone marrow cultures and were passaged in Iscove’s medium supplemented with 10% fetal bovine serum and 10 μM recombinant murine IL-3 (12), according to published methods. Cells for radiation survival curves were utilized from cultures that had been passaged for at least 10 weeks *in vitro*.

Bone marrow stromal cell line irradiation survival curves were carried out with cells irradiated to doses between 0 and 800 cGy using a cesium gamma cell irradiator. Cells were plated in six well tissue culture plates and colonies on the adherent layer consisting of >50 cells per colony were scored at day 7. IL-3-dependent hematopoietic progenitor cell lines were irradiated in suspension culture to doses described above, but then plated into semi-solid medium culture containing 0.8% methylcellulose containing Iscove’s medium supplemented with 10 μM IL-3. Non-adherent cell-derived colonies in agar secondary culture of size greater than 50 cells were scored at day 7 (11, 12).

Plating densities for both adherent stromal and non-adherent, IL-3-dependent cells were varied such that colonies counted at day 7 were in the range of 100/dish. Statistical analysis was carried out and *p*-values <0.05 were considered statistically significant. All the radiation survival curve studies were done in triplicate and the results calculated based on the average of three experiments (11, 12).

### Assays for DNA double strand breaks

DNA double strand breaks were measured by a Comet assay using a kit method (11). Briefly, bone marrow stromal cell lines, mesenchymal stem cells, or IL-3-dependent hematopoietic progenitor cell lines (hematopoietic cells) were irradiated to doses of 0 or 5 Gy, and then cells removed from suspension culture, placed on gel to assay for the migration of DNA fragments. The migration of DNA fragments is characterized as a Comet-“Tail,” and the long “Tail” is indicative of small DNA fragments migrating far from the initial cell nucleus. At least 1000 individual cells were scored for each irradiation dose, and a mean and standard error calculated based on the length of the Comet-“Tail.”

### Assay for antioxidant stores within cells and tissues

The Trolox assay was carried out to measure antioxidant stores (11). *Fancd2*^+/+^, *Fancd2*^+/−^, and *Fancd2*^−/−^ mouse stromal and IL-3-dependent hematopoietic progenitor cell lines were irradiated to doses of 0, 5, or 10 Gy. Cells were harvested at 24 h after irradiation and snap frozen on liquid nitrogen. Cell pellets were then thawed and mechanically homogenized in cold phosphate buffered solution. Protein concentrations were standardized by Bradford assay to 1 mg/mL of protein sample, and antioxidant reductive capacity (antioxidant status) was measured using a commercial kit (Northwest Life Science Specialties, Vancouver, BC, Canada). This assay measures the antioxidant capacity of cells based on the ability of cellular antioxidants to reduce Cu++ to Cu+, which reacts with bathocuproine to form a color complex absorbing at 480–490 nm. The antioxidant capacity was compared to a standard curve generator using Trolox units and all data was, therefore, expressed as millimolar or equivalents of Trolox units (11).

### Histopathologic evaluation of irradiated tissues

Oral cavity irradiation damage was scored as percent ulceration in sections of tongue tissue removed at various times after head and neck irradiation of mice (non-anesthetized). At least five sections per animal, and at least 20 animals per experiment were analyzed. Over 1000 high power microscopic fields were scored for percent ulceration, and the results presented as the percent ulceration (9, 10). The surface area of oral cavity tissue or tongue was standardized to unirradiated control as 100% intact epithelium. The percent of the surface area on these slides that was denuded, or replaced by ulceration or damage to the surface area, was then calculated based on examination of the slides. All data are presented as “percent ulceration,” which was scored on at least 10 slides/sample.

### Antioxidant small molecule therapy using GS-nitroxide

The small molecule GS-nitroxide, JP4-039 (8, 13), and related analogs are established radioprotectors and target mitochondria (14, 15). These drugs have been compared, and JP4-039 was used in the present studies. JP4-039 is in the category of hemigramicidin-targeting of the antioxidant 4-Amino-Tempol, and is used in radiation protection and mitigation studies, because of its small size. The mitochondrial targeting sequence is the smallest in JP4-039 compared to multiple other GS-nitroxides in that class (8, 13). For *in vitro* radiation protection and mitigation experiments, the compound was administered at 10 μM, and JP4-039 was added either one hour before irradiation or immediately after irradiation and maintained in the culture plates for 7 days up to the time of scoring the colony assay.

For *in vivo* experiments, JP4-039 was suspended in a novel emulsion (F15) containing Tween detergent, which facilitates localization of the compound in the locally applied tissue (16). Visualization of the small molecule to the mitochondria of cells in culture was carried out using a fluorescent BODIPY^®^-labeled modification of JP4-039 (17). For *in vivo* experiments, JP4-039/F15 was administered in a 100 μL volume intraorally to non-anesthetized mice containing 4 mg/ml JP4-039. Control groups received F15 emulsion alone in the same 100 μL volume.

### Animal safety and IACUC regulations

For all *in vivo* experiments, animal suffering was minimized and animals were sacrificed when greater than 20% body weight was reduced or significant morbidity from irradiation was determined.

## Results

### *Fancd2*^−/−^ mice are an excellent model system for radiation sensitivity issues in head and neck irradiation

*Fancd2*^−/−^, heterozygote *Fancd2*^+/−^, and wild type controls from the same litters (*Fancd2*^+/+^) were derived from both the C57BL/6J and the FVB/N background. The advantage of having *Fancd2*^−/−^ mice from two different genetic background mouse strains added robustness to the data for any experiments purporting to show radiosensitivity of normal tissues. As shown in Table 1, summary of data from previous publications indicates that compared to the heterozygote and wild type *Fancd2*^+/+^ littermates, *Fancd2*^−/−^ mice of both strains were markedly radiosensitive. In particular, bone marrow stromal cells and hematopoietic progenitor cells show distinctly different phenotypes with respect to radiation sensitivity (11). Bone marrow stromal cells are radiosensitive (11), while hematopoietic progenitor cells are radiation resistant (11). Since intact *Fancd2*^−/−^ mice display radiosensitivity in response to total body irradiation (2), the conclusion from these studies has been that the sensitivity of the microenvironment (mesenchymal stem cells, bone marrow stromal cells) communicates to hematopoietic cells the phenotype of radiosensitivity of the organ of the bone marrow. Current research in *Fancd2*^−/−^ mice has suggested that the profound radiosensitivity of the animals to total body irradiation is mediated through destruction of hematopoietic stem cells through DNA double strand breaks (4). Recent evidence suggests that aldehydes intrinsically produced by all cells are handled by aldehyde dehydrogenase-2, and when this gene is also deleted from *Fancd2*^−/−^ mice, the animals become profoundly radiosensitive (4, 18); however, this data measuring hematopoietic stem cells in irradiated mice does not answer the question of whether the lesion is mediated through hematopoietic cells, bone marrow stromal cells, or both cell phenotypes. The data presented in cell culture of distinct separated populations of cells (19) suggests that the mechanism is indirect and through the stromal cells of the microenvironment. The radiosensitivity was documented by either increased percent ulceration scoring at day 5 after single fraction irradiation, scoring at day 5 after fractionated irradiation, or in the case of the *Fancd2*^−/−^ (FVB/N) mouse model dose response curves were carried out delivering doses ranging from 24 to 30 Gy. In both model systems and both background strains, there was uniform radiosensitivity of the *Fancd2*^−/−^ genotype. Table 1 summarizes the results from two recent publications demonstrating the radiosensitivity of *Fancd2*^−/−^ mice, including experiments using single fraction, fractionated irradiation, and dose response curves. Of interest, the day 2 scoring after irradiation did not allow a significant variation between mouse genotypes; however, by day 5, ulceration was well-established and was more significant in the *Fancd2*^−/−^ mice (19).

**Table 1:** **Radiobiology of *Fancd2*^−/^^−^ (Fanconi anemia) mice and bone marrow-derived cell lines**.

Mouse strain	Total body irradiation	Longevity of hematopoiesis in long-term marrow cultures	Marrow mesenchymal stem cells	Marrow hematopoietic progenitor cells	Reference
C57BL/6J (*Fancd2*^−/−^)	Sensitive	Decreased	Radiosensitive	Radioresistant	(2, 11)
C57BL/6J (*Fancd2*^+/−^)	Intermediate	Intermediate	Intermediate	Intermediate	(2, 11)
C57BL/6J (*Fancd2*^+/+^)	Baseline	22 weeks	Baseline	Baseline	(2, 11)
FVB/N (*Fancd2*^−/−^)	Sensitive	Unknown	Radiosensitive	Radioresistant	(4, 19)
FVB/N (*Fand2*^+/−^)	Intermediate	Unknown	Intermediate	Intermediate	(19)
FVB/N (*Fancd2*^+/+^)	Baseline	Unknown	Baseline	Baseline	(19)

### Administration of the small molecule reactive oxygen species scavenger JP4-039 ameliorates irradiation-induced toxicity *in vivo*

**Figure 1:**
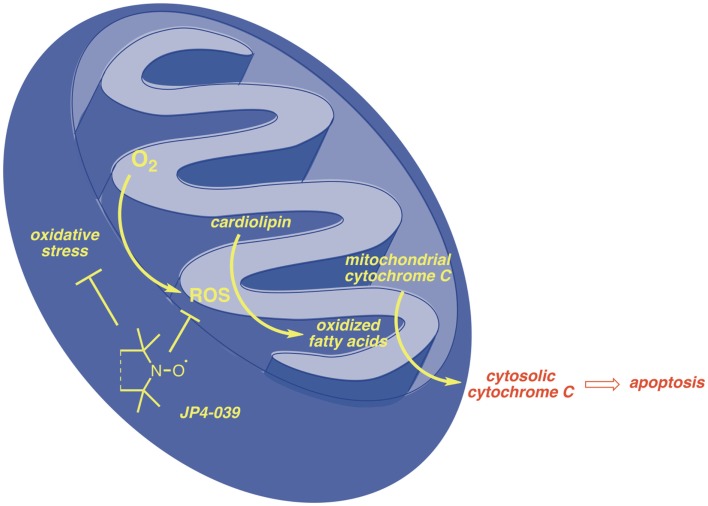
**Target of JP4-039 is the mitochondrial mechanism of irradiation-induced apoptosis**.

In both C57BL/6J and FVB/N background mouse strains, administration of JP4-039/F15 significantly ameliorated irradiation-induced mucosal ulceration, measured as tongue ulceration (Table 2). In the C57BL/6J background mouse strain, an additional control group of F15 emulsion alone showed no difference from irradiation alone control and documented the effective radiation protective effect of JP4-039. In a four fraction irradiation experiment delivering 8 Gy daily with JP4-039/F15 administered immediately before each irradiation fraction, significant radioprotection was found in *Fancd2*^−/−^ as well as heterozygote and *Fancd2*^+/+^ mice (C57BL/6J background) (Table 2). The lack of significant reduction in mucosal ulceration (tongue ulceration) in mice that had received the liposomal emulsion alone, F15, documents the radioprotective effect of the small molecule, JP4-039. Figure 1 demonstrates the possible site of action of JP4-039. JP4-039 is a mitochondrial-targeted nitroxide shown in the figure in the lower left portion of the cartoon demonstration of mitochondria (21). The nitroxide response to the oxidative stress induced in the mitochondria is both direct by formation of free radicals inside the mitochondrial intermembrane space, but also by diffusion of free radicals across the mitochondrial membrane. Radical oxygen species (ROS) come also from nuclear or cytosolic-induced superoxide and hydrogen peroxide.

**Table 2:** **Effect of GS-nitroxide (JP4-039) on radiation biology of *Fancd2*^−/^^−^ mice, tissues, and cell lines**.

Mouse strain	Total body radiation	Oral cavity	Hematopoiesis in long-term cultures	Bone marrow mesenchymal stem cells	Bone marrow hematopoietic progenitor cells	Reference
C57BL/6NHsd (JP4-039)	Increased survival	Protected	Increased	Made radioresistant	Increased resistance	(8, 16, 20)
C57BL/6J Fancd2^−/−^ + JP4-039	Unknown	Protected	Unknown	Made radioresistant	No effect	(11)
C57BL/6J Fancd2^+/−^ + JP4-039	Unknown	Unknown	Not tested	Unknown	Made radiosensitive	(11)
FVB/N Fancd2^+/+^ + JP4-039	Unknown	Protected	Not tested	Made radioresistant	Unknown	(19)
FVB/N (Fancd2^−/−^) + JP4-039	Unknown	Protected	Not tested	Made radioresistant	Unknown	(19)
FVB/N Fancd2^+/−^ + JP4-039	Unknown	Unknown	Not tested	Unknown	Unknown	(19)

**Figure 2:**
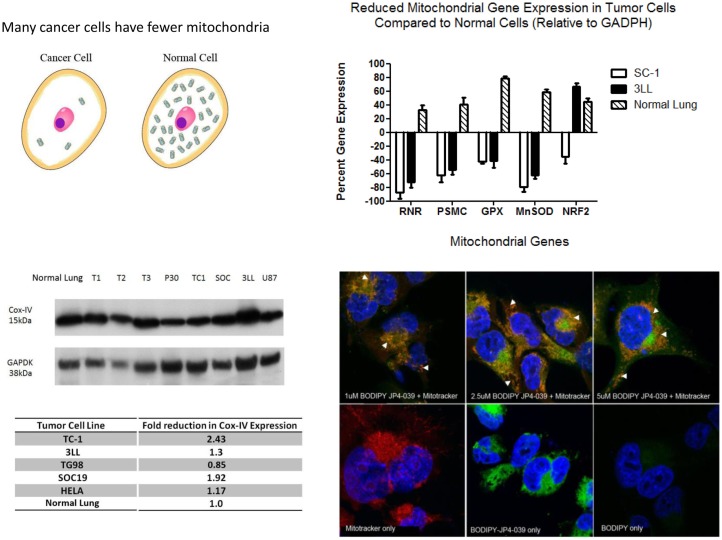
**How JP4-039 protects normal tissue**.

Free radicals have been shown to oxidize fatty acids within the mitochondria, particularly cardiolipin, but also to convert cytochrome C into a peroxidase, which can further oxidize cardiolipin (22, 23). Mitochondrial cytochrome C is tightly bound to cardiolipin and after cardiolipin oxidation is released into the cytosol. Cytochrome C leaks from the mitochondrial membrane into the cytosol. Cytosolic cytochrome C initiates apoptosis. Figure 1 demonstrates that targeting of nitroxide to the mitochondria by JP4-039 increases the capacity of the molecule to neutralize free radicals within the mitochondria, reduce the formation of oxidized cardiolipin, and reduce the capacity of the mitochondrial membrane to leak cytochrome C. The mitochondria are clearly involved in irradiation apoptosis. A summary of the currently understood mechanism of action of JP4-039 as a normal tissue radioprotector even in the setting of an in-field tumor is shown in Figure 2. Figure 2 demonstrates in four panels the proposed mechanism of action of JP4-039. In the upper left panel, cancer cells are shown as having fewer mitochondria compared to normal cells. This is confirmed by recent studies (lower left panel) with five different tumor cell lines including the mouse head and neck cancer cell line TC1, Lewis lung carcinoma (3LL), and human tumor cell lines TG98, SOC19, and HELA. All are compared to normal mouse lung with respect to density of mitochondria per weight of a cell pack. Standardizing to normal lung with a 1.0 number for density of mitochondria based on Cox-IV as a representative mitochondrial protein, standardized to GADPH, for four of the five lines (exception being TG98) shows significant reduction in mitochondria based on Cox-IV expression. As shown in the upper right hand panel of Figure 2, reduced mitochondrial gene expression as measured by RT-PCR is also documented in SC-1 (an oral squamous cell cancer line) and 3LL mouse tumor lines with respect to four mitochondrial marker RNA moieties. Only Nrf2 showed a relative similarity to normal lung in 3LL cells. The lower right hand panel of Figure 2 is relevant to the Fanconi D2^−/−^ genotype. In this figure, cells in culture were incubated with JP4-039 labeled with the BODIPY fluorochrome (17). Mitotracker localizes mitochondria, and this is shown in orange in the lower left hand panel. The BODIPY green color when added alone is shown in the far right panel lower to be diffusely seen throughout the cytoplasm. However, when attached to the JP4-039, there is localization of BODIPY to the mitochondria (upper panels). Combining the Mitotracker with the BODIPY signal shows mitochondrial localization of BODIPY–JP4-039 to the mitochondria. White arrows in the upper three panels show mitochondrial localization in a dose response curve of 1, 2, and 5 μM of the BODIPY–JP4-039 going from left to right in the upper photographs of the lower right hand panel of Figure 2. By preventing cytochrome C release from the mitochondria, JP4-039 reduces irradiation-mediated normal tissue apoptosis (15, 24).

### Oral cavity tissues are radioprotected by JP4-039, as are bone marrow stromal cells from the *Fancd2*^−/−^ background

Radiation survival curves were carried out using clonal cell lines of bone marrow stromal cells and also hematopoietic progenitor cells dependent upon IL-3, using cells derived from each of the three genotypes and each of the two background mouse strains. A recent publication documents the radiosensitivity of *Fancd2*^−/−^ bone marrow stromal cells (11).

### Three different lines of evidence suggest that the radiosensitivity of the oral cavity epithelium follows that of bone marrow stromal cells and as such is typical of mesenchymal stem cells

Adherent cells from a Fancd2^−/−^ patient were radiosensitive, and when the Fancd2 gene was re-expressed in these cells, radioresistance was restored in clonogenic survival curve assays, as well as in assays for DNA strand breaks using the Comet assay and for depleted antioxidant stores using the Trolox assay (17). In these studies with human cell lines, JP4-039 was radioprotective when added to the Fancd2^−/−^ human cells (17). In two different mouse strains, both FVB/N and C57BL/6J, *Fancd2*^−/−^ bone marrow stromal cells were radiosensitive compared to those from heterozygote or *Fancd2*^+/+^ wild type genotypes, and the radiosensitive stromal cells were significantly protected by addition of JP4-039 prior to irradiation in culture (11).

Hematopoietic cell lines growing in IL-3 culture for several months, and derived from *Fancd2*^−/−^ long-term bone marrow cultures, were paradoxically radioresistant. In experiments with both the C57BL/6J and the FVB/N background mouse strain-derived long-term bone marrow cultures, *Fancd2*^−/−^ hematopoietic cells showed a greater shoulder on the radiation survival curve in clonogenic survival assay *in vitro* (11, 19).

Most carcinomas of the head and neck region are squamous cell in histopathology. Squamous cell tumors and cell lines have fewer mitochondria due to hypoxia and reduced requirement for oxidative metabolism. Mitochondrial targeting GS-nitroxides protect cells with increased numbers of mitochondria, which advantages normal cells (Figure 2). Total bone marrow radiosensitivity in *Fancd2*^−/−^ mice might be explained based on the radiation sensitivity of stromal cells, but not hematopoietic cells (11). Bone marrow stromal cells, which are radiosensitive, are radioprotected by addition of JP4-039 (11). In the background irradiation control condition, stromal cells release humoral factors, which are toxic to hematopoietic cells and may abrogate the association/attachment of hematopoietic cells with their stroma. Thus, the radiosensitivity of the hematopoietic stem cell (niche) might overpower intrinsic radioresistance of hematopoietic cells. Prior studies have demonstrated that fresh hematopoietic progenitor cells for the colony forming unit granulocyte macrophage (CFU-GM) are radioresistant in the same *in vitro* clonogenic survival curve assay, indicating that it is not the IL-3-dependent cell line phenotype, which confers radioresistance (11).

*Fancd2*^−/−^ mice are radiosensitive to total body irradiation (2) and the totipotential reconstituting bone marrow stem cells from *Fancd2*^−/−^ mice have been shown to be relatively ineffective for repopulating total body irradiated recipients (4).

The question of whether systemic administration of JP4-039 can be radioprotective for total body irradiated *Fancd2*^−/−^ mice have not yet been investigated. Successful radioprotection by systemic administration of JP4-039, would complement the current reference studies on head and neck tissue radioprotection by local administration of JP4-039/F15, and would further strengthen the argument that the oral cavity tissues are more representative of bone marrow stromal cells than hematopoietic stem cells (11, 19).

### Recent studies show that mitochondrial-targeted small molecule radioprotection of *Fancd2*^−/−^ (FVB/N) mouse oral cavity tissues does not abrogate effective radio-controllability of orthotopic tumors

In experiments treating orthotopic tumors derived from the TC1 squamous cell carcinoma cell line (derived from C57BL/6J mice), single fraction or fractionated irradiation of mice with palpable tumors was successful in *Fancd2*^−/−^, heterozygote, and control *Fancd2*^+/+^ mice. In control tumor bearing mice, tumor size increased similarly in all three mouse strains, and a single fraction of 28 or 8 Gy times four in a four fraction experiment resulted in similar tumor radio-controllability and similar tumor size reduction independent of mouse strain genotype. However, in these recent experiments, we documented the greater radiosensitivity of [*Fancd2*^−/−^ (C57BL/6)] oral cavity tissue and amelioration of that radiosensitivity in mice receiving JP4-039/F15, even in the presence of orthotopic tumors.

The radiation survival curve of TC1 tumor cells *in vitro* was not altered by JP4-039 administration prior to or after irradiation. Given that JP4-039 has been demonstrated to localize to the mitochondria (17) and act by stabilization of antioxidant stores, stabilization of the mitochondrial membrane and prevention of irradiation-induced apoptosis (17), the failure to achieve these therapeutic goals with tumor cells *in vitro* and absence of radiation modification *in vivo* by administration of JP4-039 in F15 emulsion, suggests that the altered redox status of squamous cell carcinomas in cell lines *in vitro* or in tumors *in vivo*, may facilitate the normal tissue radioprotective action of the small molecule JP4-039 even in the setting of a DNA repair defect.

## Discussion

Clinical radiotherapy is complicated in some patients due to their clinical normal tissue radiosensitivity. Most radiation oncologists observe that 10% of their patients develop acute radiation side effects early, and in some cases, with greater intensity and longer duration, despite administration of palliative drugs, than other patients. Since most of these clinically radiosensitive patients do not have a documented genetic defect that could explain their radiosensitivity (1), most radiation oncologists observe the radiosensitivity and treat side effects at an earlier time, and continue the treatments throughout the radiotherapy course. Often in such patients, a treatment break or reduction in total dose, fraction size, or treated volume is necessary to minimize toxicity. Radioprotective agents tested in the clinic have been applied uniformly over populations of patients with no prior knowledge of who might develop side effects earlier. Such agents include Amifostine, Palifermin, and GM-CSF (1). They have achieved some success in local administration, particularly in attempts to palliate a toxicity of head and neck irradiation. In those individuals in whom a genetic cause of radiosensitivity to clinical radiotherapy is known, Fanconi anemia patients, additional care is taken before initiation of irradiation to minimize or prevent side effects. The concern for extreme toxicity of head and neck irradiation in Fanconi anemia patients motivates some clinicians to avoid irradiation in the management of such patients, reserving the therapeutic approach to surgery alone. Local regional recurrence in such patients, which may require radiotherapy, is often also restricted, and secondary surgical procedures are usually undertaken (5).

Fanconi anemia represents a particularly important diagnostic category for the testing of potential therapeutic benefits of radiation protective agents (6, 7). Susceptibility to DNA double strand breaks and irradiation-induced cell death as well as the established background propensity of these patients for bone marrow failure and secondary malignancy, makes this patient population particularly vulnerable to radiotherapy toxicity. Regrettably, the high incidence of epithelial cancers in these patients, both those surviving therapeutic bone marrow transplantation, and those not transplanted, makes it necessary to design radioprotective strategies for normal tissues in attempts to offer these patients, the same chance of long-term local control and a cure that is offered to other patient groups (5, 25–29).

For our studies, we have taken advantage of the robustness of the animal model system for *Fancd2*^−/−^ mice (2, 3). The homozygous recombinant deletion genotype is available in two different background mouse strains, C57BL/6J and FVB/N, making observations more convincing, if conserved between the two mouse strains. Recent studies had documented the radiosensitivity of *Fancd2*^−/−^ mice, specific tissues and organs from these animals, and bone marrow stromal cells established from long-term marrow culture. Radioprotection of cells in culture, and local tissues of the head and neck region by administration of an ROS scavenging small molecule (JP4-039) has been documented in cells and tissues from both mouse strains. These results provide strong evidence that an intrinsic DNA repair defect, documented in *Fancd2*^−/−^ mice, can be overcome with respect to therapeutic radiation by administration of a mitochondrial-targeted radioprotectant. These data further suggest that the initiation of radiation-induced apoptosis, which occurs in the nucleus with DNA double strand breaks, may not necessarily be fatal if the downstream effects of this initiation can be countered. The mitochondria have been shown to be a critical target for radiation protection given the strong association of mitochondrial-mediated apoptosis with ionizing irradiation (12, 17, 20, 30). Previous studies had demonstrated that initial DNA strand breaks in the nucleus are followed by rapid activation of DNA repair proteins, first ATM phosphorylation, activation of the Fanconi pathway, associated molecules including BRCA1, BRCA2, RAD51, and RAD52, and at the same time activation of stress-associated protein kinases, p53 and p21, which migrate from the nucleus to the mitochondria. At the level of the mitochondria, within minutes of irradiation of cells in culture, a cascade of events associated with specific cardiolipin–lipid peroxidation and disassociation from cytochrome C leads to mitochondrial membrane permeability and release of cytochrome C into the cytoplasm, where the caspase activation system rapidly triggers apoptosis (15, 22) (Figure 1). Preventing mitochondrial membrane permeability by targeting the oxidative stress responses prior to that permeability, and specifically ameliorating lipid peroxidation, can be achieved by mitochondrial targeting of ROS scavengers, namely JP4-039 and other related GS-nitroxides (31–33). Whether this paradigm would hold in radiosensitive cells, when the radiosensitivity is based on a known defect in the DNA repair response, has been unknown. Recent studies with *Fancd2*^−/−^ mice in two different background strains, document the radioprotective capacity of JP4-039 for cells *in vitro* (11) and for animal tissues *in vivo* (19). The available evidence suggests that patients with intrinsic radiosensitivity, where the phenomenon is based on a defect in DNA repair, can be protected by the administration of a mitochondrial-targeted antioxidant.

Studies with *Fancd2*^−/−^ mice have led to another exciting observation with respect to the radiobiology of tissue damage. *Fancd2*^−/−^ (FVB/N) mice demonstrated suppression of bone marrow colony forming progenitor cells at a greater magnitude after head and neck irradiation, than did heterozygote littermates or *Fancd2*^+/+^ control mice. That the suppression of bone marrow colony forming cells was also ameliorated by administration of head and neck localized JP4-039/F15 suggests that the humoral mediators, through the circulation, which caused the “abscopal” or “bystander” effect, may be more readily identified in these interesting *Fancd2*^−/−^ mice (19, 21, 23, 25–29, 32, 33).

Further studies with *Fancd2*^−/−^ mice should be helpful in defining many radiobiologic parameters associated with tissue, organ, and organ system responses to therapeutic fractionated irradiation in an intrinsically radiosensitive microenvironment. Furthermore, radiobiologic studies in tumors bearing *Fancd2*^−/−^ and *FancG*^−/−^ mice may lead to the clinical translation of JP4-039/F15 as a potential radioprotectant during radiotherapy of Fanconi anemia patients, or other patients with genetic defects in DNA repair.

## Conflict of Interest Statement

The authors declare that the research was conducted in the absence of any commercial or financial relationships that could be construed as a potential conflict of interest.

## References

[1] Hall EJ Giaccia AJ Radiology for the Radiologist. 6th ed Philadelphia, PA: Lippincott Williams & Wilkins (2006).

[2] Parmar K Kim J Sykes SM Shimamura A Stuckert P Zhu K Hematopoietic stem cell defects in mice with deficiency of Fancd2 or Usp 1. Stem Cells (2010) 28:1186–95 10.1002/stem.437 20506303 PMC2910804

[3] Park JW Pitot HC Strati K Spardy N Duensing S Grompe M Deficiencies in the Fanconi anemia DNA damage response pathway increase sensitivity to HPV-associated head and neck cancer. Cancer Res (2010) 70:9959–68 10.1158/0008-5472.CAN-10-1291 20935219 PMC2999655

[4] Langevin F Crossan GP Rosado IV Arends MJ Patel KJ Fancd2 counteracts the toxic effects of naturally produced aldehydes in mice. Nature (2011) 475:53–60 10.1038/nature10192 21734703

[5] Alter BP Fanconi’s anemia and malignancies. Am J Hematol (1996) 53:99–110 10.1002/(SICI)1096-8652(199610)53:23.3.CO;2-M 8892734

[6] Kee Y D’Andrea A Molecular pathogenesis and clinical management of Fanconi anemia. J Clin Invest (2012) 122:3799–806 10.1172/JCI58321 23114602 PMC3484428

[7] D’Andrea AD Grompe M Molecular biology of Fanconi anemia: implications for diagnosis and therapy. Blood (1997) 90:1725–36 9292505

[8] Goff JP Epperly MW Dixon T Wang H Franicola D Shields D Radiobiologic effects of GS-nitroxide (JP4-039) in the hematopoietic syndrome. In vivo (2011) 25:315–24 21576404 PMC3202418

[9] Guo H Seixas-Silva JA Jr Epperly MW Gretton JE Shin DM Bar-Sagi D Prevention of irradiation-induced oral cavity mucositis by plasmid/liposome delivery of the human manganese superoxide dismutase (MnSOD) transgene. Radiat Res (2003) 159:361–70 10.1667/0033-7587(2003)159[0361:PORIOC]2.0.CO;2 12600239

[10] Epperly MW Wegner R Kanai AJ Kagan V Greenberger EE Nie S Irradiated murine oral cavity orthotopic tumor antioxidant pool destabilization by MnSOD-plasmid liposome gene therapy mediates tumor radiosensitization. Radiat Res (2007) 267:289–97 10.1667/RR0761.1 17316075

[11] Berhane H Epperly MW Goff J Kalash R Cao S Franicola D Radiobiologic differences between bone marrow stromal and hematopoietic progenitor cell lines from Fanconi anemia (Fancd2-/-) mice. Radiat Res (in press). 24397476 10.1667/RR13405.1 PMC3970166

[12] Epperly MW Gretton JE Sikora CA Jefferson M Bernarding M Nie S Mitochondrial localization of copper/zinc superoxide dismutase (Cu/ZnSOD) confers radioprotective functions *in vitro* and *in vivo*. Radiat Res (2003) 160:568–78 10.1667/RR3081 14565825

[13] Gokhale A Rwigema JC Epperly MW Glowacki J Wang H Wipf P Small molecule GS-nitroxide and MnSOD gene therapy ameliorate ionizing irradiation-induced delay in bone wound healing in a novel murine model. In vivo (2010) 24:377–86 20668303 PMC2916688

[14] Fink MP Macias CA Xiao J Tyurina YY Jiang J Belikova N Hemigramicidin-TEMPO conjugates: novel mitochondria-targeted antioxidants. Biochem Pharmacol (2007) 74:801–9 10.1016/j.bcp.2007.05.019 17601494

[15] Jiang J Belikova NA Hoye AT Zhao Q Epperly MW Greenberger JS A mitochondria-targeted nitroxide/hemi-gramicidin S conjugate protects mouse embryonic cells against gamma irradiation. Int J Radiat Oncol Biol Phys (2008) 70:816–25 10.1016/j.ijrobp.2007.10.047 18262096 PMC2527544

[16] Epperly MW Goff JP Li S Gao X Wipf P Dixon T Intraesophageal administration of GS-nitroxide (JP4-039) protects against ionizing irradiation-induced esophagitis. In vivo (2010) 24:811–21 21164038 PMC3521523

[17] Bernard ME Kim H Berhane H Epperly MW Franicola D Zhang X GS-nitroxide (JP4-039) mediated radioprotection of human Fanconi anemia cell lines. Radiat Res (2011) 176:603–12 10.1667/RR2624.1 21939290 PMC3209664

[18] Garaycoechea JI Crossan GP Langevin F Daly M Arends MJ Patel KJ Genotoxic consequences of endogenous aldehydes on mouse haematopoietic stem cell function. Nature (2012) 489:571–8 10.1038/nature11368 22922648

[19] Berhane H Amelioration of irradiation induced oral cavity mucositis and distant bone marrow suppression in Fanconi anemia *Fancd2^-/-^* (FVB/N) mice by intraoral GS-nitroxide. Radiat Res (in press). 10.1667/RR13633.1 PMC4101533 24932534

[20] Goff JP Shields DS Wang H Skoda EM Sprachman MM Wipf P Evaluation of ionizing irradiation protectors and mitigators using clonogenic survival of human umbilical cord blood hematopoietic progenitor cells. Exp Hematol (2013) 41:957–66 10.1016/j.exphem.2013.08.001 23933481 PMC3834150

[21] Rajagopalan MS Gupta K Epperly MW Franicola D Zhang X Wang H The mitochondria-targeted nitroxide JP4-039 augments potentially lethal irradiation damage repair. In vivo (2009) 23:717–26 19779106 PMC2899481

[22] Kagan VE Bayir HA Belikova NA Kapralov O Tyurina YY Tyurin VA Cytochrome c/cardiolipin relations in mitochondria: a kiss of death. Free Radic Biol Med (2009) 46:1439–53 10.1016/j.freeradbiomed.2009.03.004 19285551 PMC2732771

[23] Samhan-Arias AK Ji J Demidova OM Sparvero LJ Feng W Tyurin V Oxidized phospholipids as biomarkers of tissue and cell damage with a focus on cardiolipin. Biochim Biophys Acta (2012) 1818:2413–23 10.1016/j.bbamem.2012.03.014 22464971 PMC3398793

[24] Jiang J Stoyanovsky DA Belikova NA Tyurina YY Zhao Q Tungekar MA A mitochondria-targeted triphenylphosphonium-conjugated nitroxide functions as a radioprotector/mitigator. Radiat Res (2009) 172:706–14 10.1667/RR1729.1 19929417 PMC2804962

[25] Greenberger JS Epperly MW Radioprotective antioxidant gene therapy: potential mechanisms of action. Gene Ther Mol Biol (2004) 8:31–44

[26] Greenberger JS Epperly MW Pleiotropic stem cell and tissue effects of ionizing irradiation protection by MnSOD-plasmid liposome gene therapy. In: Columbus F, editor. Progress in Gene Therapy. Hauppauge, NY: Nova Science Publications (2005). p. 110–8

[27] Greenberger JS Radioprotection. In vivo (2009) 23:323–36 19414422 PMC2981866

[28] Tarhini AA Belani CP Luketich JD Argiris A Ramalingam SS Gooding W A phase I study of concurrent chemotherapy (Paclitaxel and Carboplatin) and thoracic radiotherapy with swallowed manganese superoxide dismutase (MnSOD) plasmid liposome (PL) protection in patients with locally advanced stage III non-small cell lung cancer. Hum Gene Ther (2011) 22:336–43 10.1089/hum.2010.078 20873987 PMC3057216

[29] Kalash R Berhane H Goff J Houghton F Epperly MW Dixon T Thoracic irradiation effects on pulmonary endothelial compared to alveolar type II cells in fibrosis prone C57BL/6NTac mice. In vivo (2013) 27:291–8 23606683 PMC3783952

[30] Epperly MW Melendez JA Zhang X Nie S Pearce L Peterson J Mitochondrial targeting of a catalase transgene product by plasmid liposomes increases radioresistance *in vitro* and *in vivo*. Radiat Res (2009) 171:588–95 10.1667/RR1424.1 19580494 PMC2762783

[31] Hoye AT Davoren JE Wipf P Fink MP Kagan VE Targeting mitochondria. Acc Chem Res (2008) 41:87–97 10.1021/ar700135m 18193822

[32] Greenberger JS Clump D Kagan V Bayir H Lazo JS Wipf P Mitochondrial targeted small molecule radiation protectors and radiation mitigators. Front Radiat Oncol (2012) 1:1–12 10.3389/fonc.2011.00059 22655254 PMC3356036

[33] Kalash R Epperly MW Goff J Dixon T Sprachman MM Zhang X Amelioration of irradiation pulmonary fibrosis by a water-soluble bi-functional sulfoxide radiation mitigator (MMS350). Radiat Res (2013) 180:474–90 10.1667/RR3233.1 24125487 PMC3894523

